# Measurement of Quality of Life in Patients with Mycosis Fungoides/Sézary Syndrome Cutaneous T-Cell Lymphoma: Development of an Electronic Instrument

**DOI:** 10.2196/11302

**Published:** 2019-01-07

**Authors:** Stacey McCaffrey, Ryan A Black, Mitchell Nagao, Marjan Sepassi, Gaurav Sharma, Susan Thornton, Youn H Kim, Julia Braverman

**Affiliations:** 1 PatientsLikeMe, Inc Cambridge, MA United States; 2 Nova Southeastern University Fort Lauderdale, FL United States; 3 Actelion (US), Inc South San Francisco, CA United States; 4 Cutaneous Lymphoma Foundation Birmingham, MI United States; 5 Stanford University Stanford, CA United States

**Keywords:** quality of life, Rasch, patient-reported outcome, cutaneous T-cell lymphoma, mycosis fungoides, Sézary syndrome

## Abstract

**Background:**

Although the quality of life (QoL) plays an important role in treatment decision making and clinical management of mycosis fungoides (MF) or Sézary syndrome (SS) subtypes of cutaneous T-cell lymphomas (MF/SS-CTCLs), an MF- or SS-specific measure of QoL does not exist.

**Objective:**

The objective of this research was to develop and validate the first QoL instrument for MF/SS-CTCL using a patient-centered approach.

**Methods:**

A conceptual framework for the MF/SS-CTCL QoL was developed through a literature review and interviews with key opinion leaders. Concept elicitation with patients was utilized to refine the conceptual model and generate preliminary items. The items were then revised based on qualitative and quantitative feedback obtained through cognitive debriefing surveys and interviews with patients. Next, participants (N=126) completed the preliminary MF/SS-CTCL QoL and a comparator measure of health-related QoL (Skindex-29) through the PatientsLikeMe Open Research Exchange. The MF/SS-CTCL QoL was completed again 5 days later by 66 participants for the purposes of evaluating test-retest reliability. The MF/SS-CTCL QoL was finalized based on results from an empirical evaluation, which included both classical and modern test theory approaches. Specifically, this included evaluation of (1) the optimal item response theory measurement model; (2) item fit; (3) unidimensionality; (4) rating scale performance; (5) reliability; (6) test information (precision); (7) person-to-item map; (8) convergent and discriminant validity; and (9) presence of bias via differential item function.

**Results:**

Results from the comprehensive psychometric evaluation utilizing a Rasch-Grouped Rating Scale model yielded a final 12-item instrument. The rating scale functioned as expected, and the instrument exhibited adequate person reliability (.87), good to excellent test-retest reliability (*r*=.89, *P*<.001), high levels of measurement precision, and good person-to-item targeting. The correlation between the MF/SS-CTCL QoL and the Skindex-29 (*r*=.852, *P*<.001) was significantly greater than the correlation between the MF/SS-CTCL QoL and syndrome stage (*r*=.260, *P*<.001), providing support for convergent and discriminant validity. Items did not show significant bias based on gender, age, or race. Rasch scores were converted to scaled scores with qualitative descriptive categories for ease of interpretation.

**Conclusions:**

Empirical evaluation demonstrated strong evidence of excellent psychometric properties. Utilizing a patient-centered measure development approach ensures that this QoL instrument captures the information that is most meaningful and clinically relevant to patients.

## Introduction

Mycosis fungoides (MF) and its leukemic variant Sézary syndrome (SS) represent approximately 65% of the cases of cutaneous T-cell lymphoma (CTCL), a class of non-Hodgkin’s lymphomas with a relapsing course over the span of decades [1,2]. For patients with MF or SS subtypes of CTCL (MF/SS-CTCL), quality of life (QoL) plays an important role in treatment decision making and clinical management of the disease. Currently, several cancer-specific (European Organisation for Research and Treatment of Cancer tools [3] and Functional Assessment of Cancer Therapy-General [4]) and skin-specific (ItchyQoL [5], Dermatology Life Quality Index [6], Skindex-29 [7], and itch visual analog scale [8]) health-related QoL instruments exist. Although clinicians often use these instruments or a combination of these instruments to estimate QoL for patients with MF/SS-CTCL, this strategy can be time consuming and burdensome for patients. These patient-reported outcome (PRO) instruments were not specifically designed to capture the unique experiences of patients living with MF/SS-CTCL. They may contain content that is irrelevant for patients with MF/SS-CTCL and fail to capture the aspects of QoL that are most meaningful for these patients, decreasing the instrument’s sensitivity in detecting important changes in QoL [9]. Therefore, a patient-centered, disease-specific PRO to measure QoL for patients with MF/SS-CTCL is urgently needed to improve the quality of care for these patients and to progress research within this clinical arena. The purpose of this study is to fill this critical gap by developing and validating the first QoL instrument specifically developed by and designed for patients with MF/SS-CTCL—the “MF/SS-CTCL QoL.” The MF/SS-CTCL QoL was developed in two broad phases: (1) instrument development and (2) psychometric evaluation. Methods and results of each phase are described separately below.

## Methods

### Instrument Development

The purpose of instrument development was to create items that comprehensively capture the different facets of QoL that are impacted by MF/SS-CTCL. Patients were closely involved in the item development process to ensure that the final instrument evaluated aspects of QoL that are most relevant and meaningful for them. Instrument development involved three primary steps: (1) creating a conceptual framework; (2) concept elicitation; and (3) cognitive debriefing. This research was approved by the New England Institutional Review Board.

#### Creating the Conceptual Framework

The conceptual framework of QoL for MF/SS-CTCL patients was developed through a literature review and interviews with key opinion leaders (KOLs). Physicians and experts in the field of cutaneous lymphomas (N=3) participated in interviews to gather information related to treatment, challenges in caring for and treating patients with MF/SS-CTCL, main concerns expressed by patients, the impact of the condition on patients’ well-being and daily functioning, use and availability of PRO instruments, and unmet needs within the research and patient care field.

Results from the literature review and KOL interviews highlighted the importance of evaluating condition-specific facets of QoL, such as physical functioning, emotional functioning, and social functioning. KOLs also indicated that two facets of QoL—coping and self-management—were absent from existing PRO measures and may be important for patients with MF/SS-CTCL. More information about KOL input and the conceptual model is available upon request.

#### Concept Elicitation

The purpose of concept elicitation was to gather patient feedback regarding their experience of living with MF/SS-CTCL and generate preliminary items. Data for concept elicitation were collected from patients through a survey conducted via the PatientsLikeMe Web-based research platform (Open Research Exchange, ORE) and follow-up interviews conducted using phone or videoconferencing. Patients were eligible to participate if they were members of PatientsLikeMe, were adults, and reported a diagnosis of MF or SS. Survey content included demographic and clinical items as well as open-ended questions pertaining to health-related QoL derived from the conceptual model. Follow-up interviews consisted of semistructured questions based on participants’ responses to the survey.

The data were coded independently by two trained raters using MAXQDA software (VERBI). The coders (a research scientist and research assistant), experienced in qualitative research and coding, were trained by the senior author (JB). The raters coded the interviews independently, and any discrepancies in codes were resolved by the senior author. The codebook was finalized after a satisfactory interrater agreement (Cohen kappa of 0.65 or greater) was reached. Content saturation was assessed across patients with a saturation table where saturation was reached when no new information was obtained through data collection [10]. The codes with best agreement and highest frequencies were selected and then grouped by themes to generate the initial items for the QoL instrument.

##### Results From Concept Elicitation

The Web-based survey was completed by 21 participants, and 10 of those participants completed a follow-up interview. The sample comprised 67% (14/21) females, all of them being white and non-Hispanic, and the average age was 55 (SD 12.39) years. Of the 21 participants, 16 (76%) reported a diagnosis of MF, 3 (14%) reported a diagnosis of SS, and 2 (10%) did not report a diagnosis. The average disease length was 10 (SD 9.50) years, with a range of <1 year to 31 years. Among all, 14 participants reported their stage of diagnosis and indicated that they had stage IA (8/21, 38%), stage IB (4/21, 19%), or stage IIB (2/21, 10%) disease.

Based on the qualitative analysis, 43 of the 60 codes developed from the coding scheme reached agreement of a Cohen kappa at or above 0.65. Saturation was reached after 15 patients, suggesting an adequate sample size. Thematic content analysis identified six major code groupings (treatment, impact on daily activities, emotional, social, coping and management, and symptoms and symptom burden), which were used to generate the 31-item preliminary version of the MF/SS-CTCL QoL (more details about the survey and results are available upon request).

#### Cognitive Debriefing

Using the same Web-based research platform (ORE) and participant inclusion criteria from the concept elicitation phase, the preliminary version of the MF/SS-CTCL QoL was administered to a sample of participants. Although a partnership between PatientsLikeMe and the Cutaneous Lymphoma Foundation (a patient-advocacy group) was made to help with patient recruitment, there was substantial overlap in participants across stages of the research study due to difficulty recruiting patients with these rare diseases. Participants were asked to complete the preliminary items and to provide specific quantitative and qualitative feedback regarding clarity or semantic ambiguity and understanding, relevance, and adequacy of each item and the response options.

##### Results From Cognitive Debriefing

Overall, 42 participants took part in cognitive debriefing. Of the 41 participants who chose to provide demographic information, approximately half were men (51%, 21/41) with an average age of 62 (SD 14.13; range 31-101) years. The majority (85%, 35/41) reported a diagnosis of MF. Their disease stage ranged from IA to IVA, with most participants reporting stage IA (39%, 16/41) or stage IB (17%, 7/41) disease.

Based on quantitative and qualitative cognitive debriefing results, changes were made to the instrument; items were removed and revised to improve clarity, a response option was added for patients who were in remission, and the recall period was changed from 7 days to 4 weeks. At this stage, the MF/SS-CTCL QoL contained 14 items.

### Psychometric Evaluation

#### Participants

Patients were eligible to participate if they were members of the Web-based community (PatientsLikeMe), adults (aged ≥18 years), and reported a diagnosis of MF or SS. Participants were recruited through the PatientsLikeMe Web-based community with support from the Cutaneous Lymphoma Foundation.

#### Data Collection

Following consent, eligible participants completed a demographic survey, the MF/SS-CTCL QoL, and the Skindex-29 [7] through the Web-based research platform. Additionally, participants were asked to complete a second administration of the MF/SS-CTCL QoL 5 days later to evaluate the stability of item functioning.

#### Measures

The Skindex-29 [7] is a commonly used and valid 29-item self-report measure that evaluates health-related QoL. Specifically, the Skindex-29 covers facets of QoL such as emotional functioning, physical functioning, and symptoms with a 4-week recall period. The preliminary 14-item version of the MF/SS-CTCL QoL required patients to rate their impairment in health-related QoL over the last 4 weeks using a 1 (not at all or never) to 5 (very much or always) Likert-type rating scale. Furthermore, 4 of the items included a sixth response option, “Does not apply (I don’t have symptoms right now).”

As part of this study, participants also completed a brief demographics survey asking them to provide information about their sex, age, race, ethnicity, diagnosis, and stage of their diagnosis.

#### Psychometric Evaluation Procedures

The empirical evaluation included determining (1) the optimal item response theory (IRT) measurement model; (2) item fit; (3) unidimensionality; (4) rating scale performance; (5) reliability; (6) test information (precision); (7) person-to-item map; (8) convergent and discriminant validity; and (9) presence of bias via differential item function (DIF). Analyses were performed in SPSS version 24 (IBM Corp) and Winsteps version 3.74.0 (Winsteps.com).

## Results

### Participants

A total of 126 patients completed the survey, and 52.4% (66/126) patients completed the second administration of the survey. Most participants were non-Hispanic (115/126, 91.3%) individuals and identified as white or Caucasian (108/126, 85.7%) and most were females (74/126, 58.7%). Participants ranged in age from 22 to 86 years, with an average age of 59 (SD 13.5) years. Of the 126 participants, 118 (93.7%) reported a diagnosis of MF and 8 (6.3%) reported a diagnosis of SS. Participants indicated that they had stage IA (56/126, 44.4%), stage IB (24/126, 19.0%), or stage II or above (22/126, 17.5%) disease and 19.0% (24/126) did not know or report their stage. The average disease length was 8 (SD 7.6) years, with a range of <1 year to 35 years. A summary of participants’ prescribed treatments is presented in Table 1.

### Item Descriptive Statistics

Item descriptive statistics are presented in Table 2. Responses of “Does not apply (I don’t have symptoms right now)” (score=0) were marked as missing and removed from analyses when calculating mean and SD.

**Table 1:** Participants’ self-reported prescribed treatments.

Prescribed treatment	Value, n (%)^a^
Topical corticosteroids	79 (62.7)
Other prescribed topical treatments	40 (31.7)
Phototherapy (psoralen and ultraviolet A, ultraviolet B)	12 (9.5)
Total-skin electron beam therapy	16 (12.7)
Local radiation therapy	14 (11.1)
Oral treatments or chemotherapy	113 (89.7)

^a^Treatment % is greater than 100% due to multiple selections being allowed.

**Table 2:** Item descriptive statistics.

Item	Minimum score	Maximum score	Mean (SD)
1. In the past 4 weeks, how much did you worry that your mycosis fungoides or Sézary syndrome may get worse?	1	5	2.74 (1.26)
2. In the past 4 weeks, how often did you feel hopeless because of having mycosis fungoides or Sézary syndrome?	1	5	2.01 (1.13)
3. In the past 4 weeks, how frustrated were you by the unpredictability of mycosis fungoides or Sézary syndrome?	1	5	2.67 (1.36)
4. In the past 4 weeks, how often did you feel depressed or sad because of mycosis fungoides or Sézary syndrome?	1	5	2.15 (1.03)
5. In the past 4 weeks, how confident did you feel about managing your mycosis fungoides or Sézary syndrome?	1	5	2.94 (1.11)
6. In the past 4 weeks, to what extent were you able to cope with the daily demands (symptom impact and management, treatment, side effects, appointments, etc) of mycosis fungoides or Sézary syndrome?	1	5	3.47 (1.27)
7. In the past 4 weeks, how severe were your mycosis fungoides or Sézary syndrome symptoms?	1	5	1.95 (1.05)
8. In the past 4 weeks, how burdensome was your mycosis fungoides or Sézary syndrome treatment?	1	5	2.20 (1.02)
9. In the past 4 weeks, how much did your mycosis fungoides or Sézary syndrome limit your daily activities (work inside and outside of the house, self-care such as cooking, cleaning, getting dressed, etc)?	1	5	1.79 (1.25)
10. In the past 4 weeks, how much did mycosis fungoides or Sézary syndrome limit your ability to wear clothes you wanted to?	1	5	2.28 (1.49)
11. In the past 4 weeks, how often did mycosis fungoides or Sézary syndrome (the condition or associated treatment) leave you too tired to work or do daily activities?	1	5	2.11 (1.20)
12. In the past 4 weeks, how much did mycosis fungoides or Sézary syndrome negatively affect your relationships with others close to you?	1	5	1.73 (1.10)
13. In the past 4 weeks, how often did you feel that others do not understand what you are going through with mycosis fungoides or Sézary syndrome?	1	5	2.67 (1.33)
14. In the past 4 weeks, to what extent did mycosis fungoides or Sézary syndrome make you feel uncomfortable being around people other than close family and friends?	1	5	1.94 (1.20)

### Identifying the Optimal Item Response Theory Measurement Model

Determining the optimal IRT model to calibrate the items was an iterative process based on empirical evidence and substantive rationale [11,12]. Since items were grouped into 2 rating scales, frequency and intensity, the Andrich-Grouped Rating Scale Model (G-RSM [12]) and Rating Scale Model (RSM [13]) were considered. Of note, the Partial Credit Model and Generalized Partial Credit Model were not considered as these models would likely produce unstable estimates due to the number of parameters to be estimated relative to the sample size.

To determine whether the rating scales for the intensity items and frequency items could be grouped, respectively, a partial credit model was used, and item characteristic curves (ICCs) were generated. These ICCs were similar within the groups (frequency and intensity). Next, a global chi-square test was performed to test whether the G-RSM significantly improved the fit above and beyond the RSM. Results revealed that the G-RSM significantly improved model fit over the RSM (χ^2^_3_=8.7; *P*=.03).

### Item Fit

Item fit was evaluated by examining item mean square infit and outfit statistics. There were 2 items that evidenced infit and outfit statistics above the commonly accepted cut-off of 1.33 [14] and were iteratively removed. Of note, these items still provide useful information about the patient experience and can be used in conjunction with the MF/SS-CTCL QoL global rating (see Multimedia Appendix 1). The remaining items evidenced adequate fit statistics and were retained for further analyses.

### Unidimensionality

The assumption of unidimensionality was evaluated via an unrotated principal components analysis on the probability scale residuals in Winsteps [15]. Although the eigenvalue suggested the possible presence of a second dimension (eigenvalue=2.2), evaluation of item content and amount of variance explained by the Rasch measurement model (62.5%) provided support that the 12 items were measuring a unidimensional construct.

### Evaluation of Rating Scale Performance

**Figure 1:**
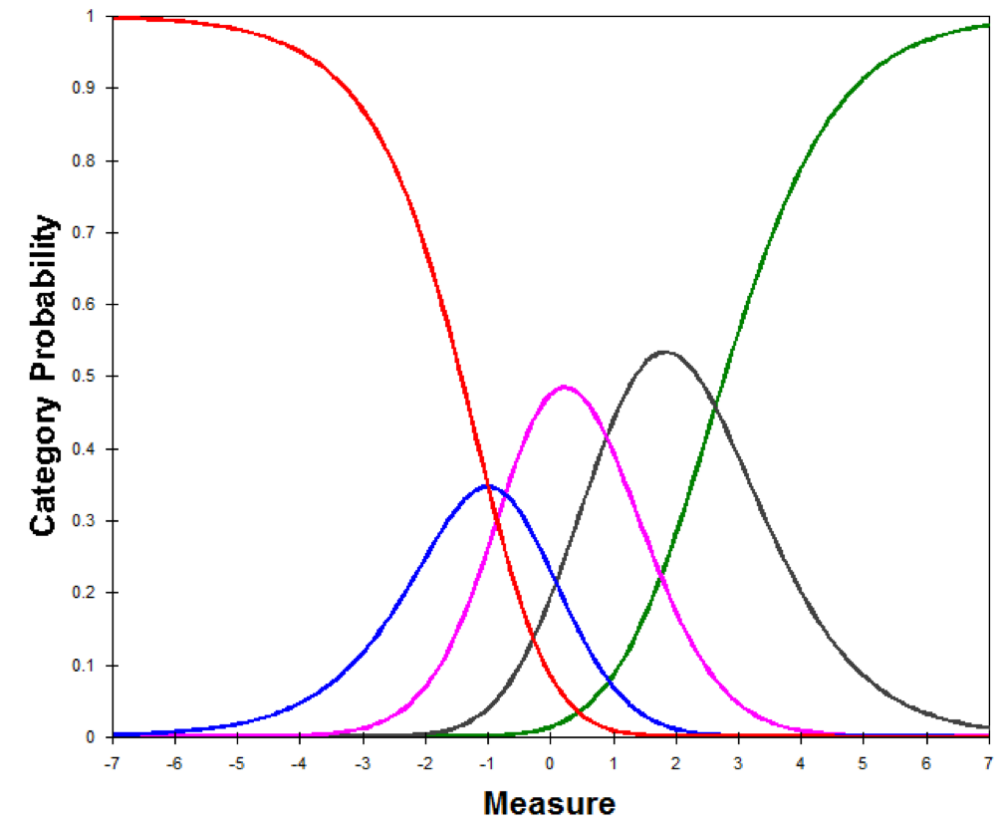
Category response curves for the frequency items.

**Figure 2:**
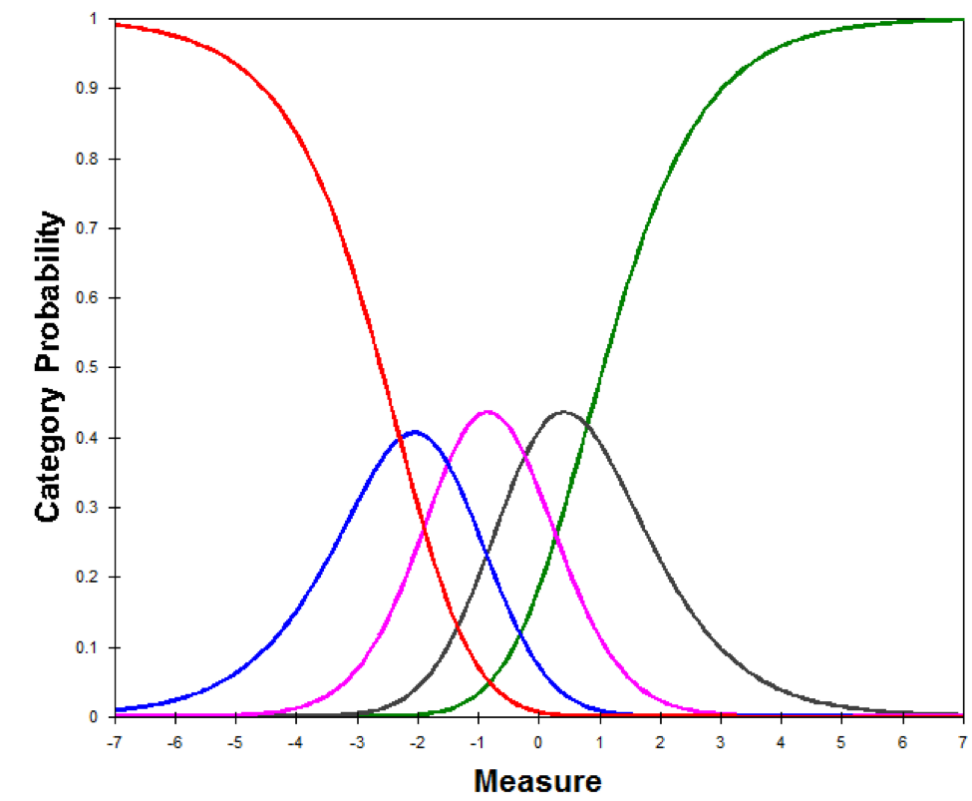
Category response curves for the intensity items.

Andrich thresholds were examined to further ensure that the rating scales for the set of intensity items and the set of frequency items were performing as expected. Thresholds were ordered, indicating that a higher interference in QoL is required to endorse a higher frequency or intensity response category (Figures 1 and 2). Figure 1 depicts the relationship between interference with QoL and response option selection for the frequency items, whereby the different color curves represent the probability of selecting one of the response options. Specifically, “never,” “rarely,” “sometimes,” “often,” and “always” are represented by the red, blue, purple, gray, and green curves, respectively. This figure shows that a higher interference in the level of QoL is required to endorse a higher frequency.

Figure 2 depicts the relationship between interference with QoL and response option selection for the intensity items, whereby the different color curves represent the probability of selecting one of the response options. Specifically, “not at all,” “a little bit,” “somewhat,” “quite a bit,” and “very much” are represented by the red, blue, purple, gray, and green curves, respectively. This figure shows that a higher interference in the level of QoL is required to endorse a higher intensity.

### Reliability

Person reliability for the 12-item scale was 0.87, suggesting that the MF/SS-CTCL QoL is able to discriminate between individuals with low and high levels of interference in their QoL. Item reliability was 0.97, which suggests that the sample was large enough to locate items on QoL. Test-retest reliability (*r*=0.89; *P*<.001), calculated through a Pearson correlation between MF/SS-CTCL QoL scores at time 1 and time 2 (5 days later) revealed good to excellent stability.

### Test Information

**Figure 3:**
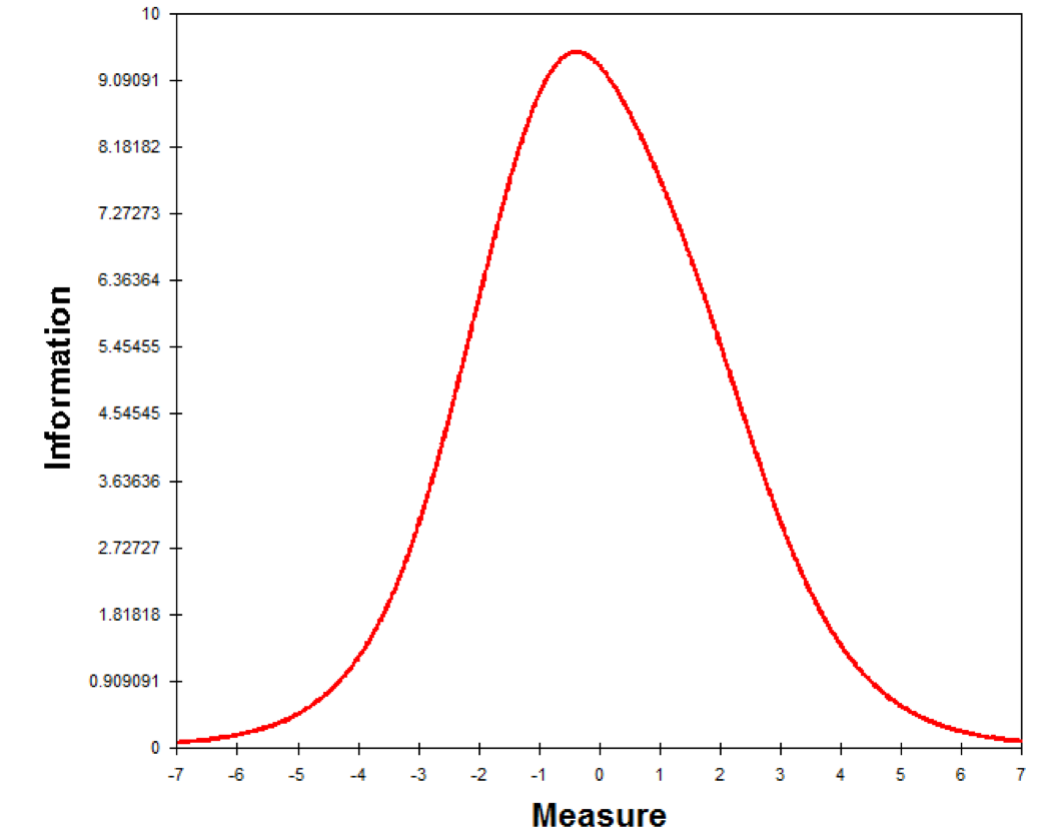
Test information curve.

A test information curve was generated to evaluate the measurement precision of the MF/SS-CTCL QoL at various levels of the latent trait (QoL). The test information curve (Figure 3) provides evidence that the amount of interference with QoL was precisely estimated and that the MF/SS-CTCL QoL is best at differentiating people who have trait levels within about 2 SDs of the mean. Figure 3 depicts the amount of information (or precision of measurement) that is provided by the MF/SS-CTCL QoL measure across the latent construct of interference with QoL.

### Person-to-Item Map

**Figure 4:**
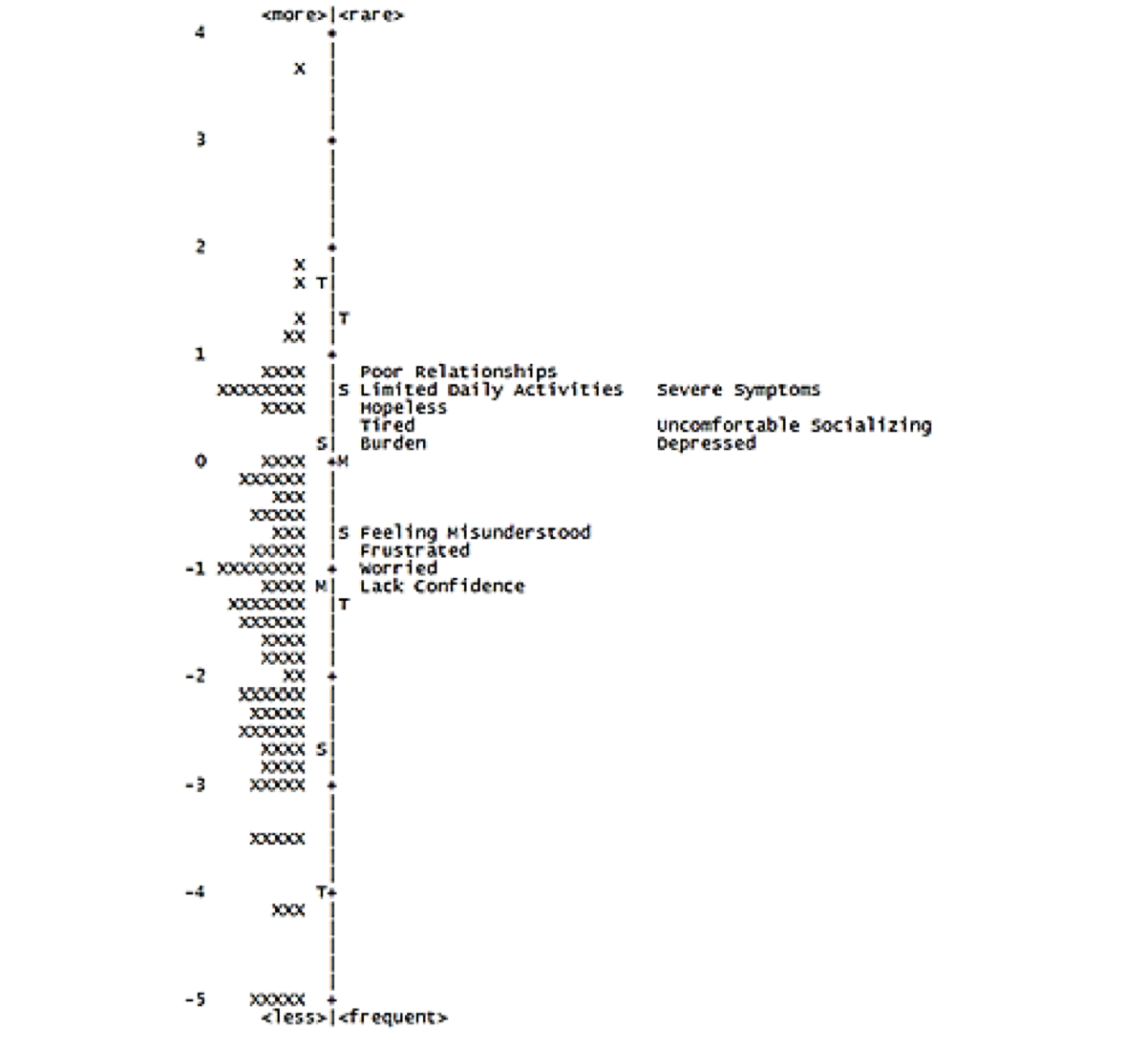
Person-to-item map.

Due to the unique properties of the Rasch model, it is possible to place both persons and items on the same interval-level scale or “ruler,” depicted using a person-to-item map (Figure 4). This map can be interpreted as a vertical ruler, with persons (depicted on the left) and items (depicted on the right) ordered in relation to their difficulty or trait level using a scale (logits) with a mean of 0 and SD of 1. For example, on the MF/SS-CTCL QoL, “In the past 4 weeks, how confident did you feel about managing your mycosis fungoides or Sézary syndrome?” and “In the past 4 weeks, how much did you worry that your mycosis fungoides or Sézary syndrome may get worse?” were found to be easier (ie, require less impairment in QoL) to endorse. On the other hand, items near the top of the person-to-item map require a higher impairment in QoL to endorse (eg, “In the past 4 weeks, how much did mycosis fungoides or Sézary syndrome negatively affect your relationships with others close to you?” and “In the past 4 weeks, how much did your mycosis fungoides or Sézary syndrome limit your daily activities, ie, work inside and outside of the house, self-care such as cooking, cleaning, getting dressed, etc?”). This map is presented in Figure 4. Overall, examination of the map suggests adequate coverage of items across much of the latent trait. However, visual inspection of the map suggests limited person-to-item targeting at lower levels of interference with quality of life.

### Convergent and Discriminant Validity

To evaluate convergent and discriminant validity, a correlation matrix of the MF/SS-CTCL QoL, the Skindex-29, and syndrome stage was constructed. It was hypothesized that the MF/SS-CTCL QoL would be significantly more positively correlated with the Skindex-29, another QoL measure (convergent validity), than with syndrome stage (discriminant validity). The correlation between the MF/SS-CTCL QoL and the Skindex-29 (*r*=0.852; *P*<.001) was significantly greater than that between the MF/SS-CTCL QoL and syndrome stage (*r*=0.260; *P*<.001), providing support for convergent and discriminant validity.

### Differential Item Function

DIF generally occurs when participants with an equal amount of the latent trait (interference in QoL) respond differently to an item [11]. DIF was assessed by gender, age, and race (white or nonwhite). DIF was considered notable if the DIF contrast estimate was >1.0 logit and significant at alpha=.05. Results revealed that the items did not have DIF at high enough levels to be considered problematic.

### Scoring the Mycosis Fungoides/Sézary Syndrome Cutaneous T-Cell Lymphoma Quality of Life

A total raw MF/SS-CTCL QoL score is calculated by adding up the patient’s total score from the 12 MF/SS-CTCL QoL items. Table 3 provides scaled scores (mean 100, SD 15) that correspond to the MF/SS-CTCL QoL total score. Although total raw scores of 10 or 11 are possible due to 2 items with the response choice “Does not apply (I don’t have symptoms right now),” these scores should not be interpreted differently from a score of 12. For scoring purposes, “Does not apply (I don’t have symptoms right now)” is scored as a 0. Furthermore, in order to score the MF/SS-CTCL QoL, each of the 12 items must be completed.

**Table 3:** Raw to scaled score conversion table.

Raw MF/SS-CTCL QoL^a^ score	Scaled score^b^
≤12^c^	62
13	74
14	80
15	84
16	87
17	89
18	91
19	93
20	94
21	96
22	97
23	98
24	100
25	101
26	102
27	103
28	104
29	105
30	106
31	107
32	108
33	109
34	110
35	111
36	112
37	113
38	114
39	115
40	116
41	117
42	118
43	119
44	120
45	121
46	123
47	124
48	125
49	126
50	128
51	129
52	131
53	133
54	135
55	137
56	139
57	143
58	147
59-60	154

^a^MF/SS-CTCL: mycosis fungoides/Sézary syndrome cutaneous T-cell lymphoma quality of life.

^b^Scaled scores were standardized on the current sample to have a mean of 100 and an SD of 15.

^c^While it is possible to obtain a raw score of 10 or 11 due to endorsing “Does not apply (I don’t have symptoms right now)” to MF/SS-CTCL QoL items, these scores should be viewed as equivalent to a 12.

**Table 4:** Qualitative descriptions of mycosis fungoides/Sézary syndrome cutaneous T-cell lymphoma quality of life scaled scores.

Scaled score	Description
62 to 89	No to low interference
91 to 105	Mild interference
106 to 117	Moderate interference
118 to 133	Substantial interference
135 to 154	Severe interference

### Mycosis Fungoides/Sézary Syndrome Cutaneous T-Cell Lymphoma Quality of Life Interpretation

The qualitative description of scaled scores, provided in Table 4, was based on evaluating the distribution of scaled scores relative to the response categories. For example, individuals with scaled scores that corresponded with an average rating of 1 (not at all or never) across the items were described as having no to low interference, individuals with scaled scores that corresponded with an average rating of 2 (a little or rarely) were described as having mild interference, and individuals with scaled scores that corresponded with an average rating of 3 (somewhat or sometimes) across the items were described as having moderate interference (Table 4).

## Discussion

### Principal Findings

This research utilized a multistage instrument development process that incorporated both qualitative and quantitative components, including (1) development of a conceptual model through literature review and input from KOLs; (2) refinement of the conceptual model and generation of preliminary items through concept elicitation with patients; (3) item revisions based on feedback from patients during cognitive debriefing; and (4) empirical testing to evaluate psychometric functioning and finalize the MS/SS-CTCL QoL. The results provide strong support for reliability and validity of the MS/SS-CTCL QoL. Specifically, results indicate that the rating scale was functioning as expected, and the 12-item MS/SS-CTCL QoL exhibited adequate person reliability, excellent test-retest reliability, high levels of measurement precision, good person-to-item targeting, and evidence of convergent and discriminant validity. Items did not evidence significant bias based on gender, age, or race.

This study used state-of-the-art modern test theory approaches, which are considered the “gold standard” in test construction methodology as they rely on stronger measurement assumptions and produce more reliable results than classical approaches [11,16]. Further, Rasch modeling allows for new items to be incorporated into the instrument without having to establish the validity of the entire bank. This advantage may be particularly important as treatments improve and disease management changes over time.

### Limitations

The sample of patients with MF who participated in this validation study was largely of those with stage I disease. Incorporating a greater number of patients who represent the more advanced stages in the item generation process may have resulted in different item content. Consequently, gathering feedback from patients with more advanced stages will likely be a critical part of future instrument refinement.

All data collected from patients during this study relied exclusively on patient report, and patient diagnosis and stage could not be verified by a licensed medical professional. Additionally, patients were recruited from the internet, potentially excluding patients who do not have internet access or those whose health or functioning may interfere with their ability to use the internet. Similarly, many of the participants for this research were recruited through PatientsLikeMe, potentially limiting the generalizability of findings. For example, members of the PatientsLikeMe Web-based community may be more conscious, engaged in their health, and comfortable sharing health information than the general population [17]. Therefore, future research may evaluate whether the items function differently among patients recruited through different sources, such as hospitals or university clinics.

Despite the partnership from the Cutaneous Lymphoma Foundation to assist with recruitment of patients with this rare disease, obtaining sample sizes adequate for each phase of measure development was challenging, and the same patients participated in several stages of the development process. Additional studies should be performed to replicate these findings. Future research might also evaluate this instrument’s ability to detect change over time as a patient’s stage, treatment, or health status changes.

Finally, evaluation of person-to-item targeting suggested that this scale may be limited in its ability to differentiate persons who may be experiencing little to no interference with QoL. However, from a clinical perspective, this is likely not problematic. That is, clinicians may be less concerned with precisely measuring interference with QoL and differentiating between patients who are very low on interference with QoL than with precisely measuring and tracking interference with QoL in patients who are experiencing some level of interference.

### Conclusions

The MF/SS-CTCL QoL is the first MF/SS-specific instrument to capture the impact of MF/SS-CTCL on patients’ health-related QoL. Incorporating the patient perspective throughout the development process likely increased the relevancy of MF/SS-CTCL QoL content for this patient population. The MF/SS-CTCL QoL was developed in partnership with the Cutaneous Lymphoma Foundation with the intention of improving care for MF/SS-CTCL patients. Therefore, the MF/SS-CTCL QoL is free for clinicians, patients, and researchers and can be downloaded free of charge from the ORE.

## Appendix

Multimedia Appendix 1 The MF/SS-CTCL QoL instrument.

## Abbreviations

CTCL: cutaneous T-cell lymphoma
DIF: differential item function
G-RSM: Grouped Rating Scale Model
ICC: item characteristic curve
IRT: item response theory
KOL: key opinion leader
MF: mycosis fungoides
ORE: Open Research Exchange
PRO: patient-reported outcome
QoL: quality of life
RSM: Rating Scale Model
SS: Sézary syndrome
