# Supplementary material for: Measurement of Quality of Life in Patients with Mycosis Fungoides/Sézary Syndrome Cutaneous T-Cell Lymphoma: Development of an Electronic Instrument
Source: J Med Internet Res. 2019 Jan 7;21(1):e11302. doi: 10.2196/11302 (PMC6329422; doi:10.2196/11302)
Supplement: Multimedia Appendix 1 [file jmir_v21i1e11302_app1.pdf]

Multimedia Appendix 1

MF/SS-CTCL QoL

Please complete these items regarding your experiences over the past 4 weeks.

In the past 4 weeks…

Q1***…how much did you worry that your mycosis fungoides or Sézary syndrome may get worse?***

1	2	3	4	5
Not at all	A little bit	Somewhat	Quite a bit	Very much

Q2. ...***how often did you feel hopeless because of having mycosis fungoides or Sézary syndrome?***

1	2	3	4	5
Never	Rarely	Sometimes	Often	Always

Q3. ***...how frustrated were you by the unpredictability of mycosis fungoides or Sézary syndrome?***

1	2	3	4	5
Not at all	A little bit	Somewhat	Quite a bit	Very much

Q4...***how often did you feel depressed or sad because of mycosis fungoides or Sézary syndrome?***

1	2	3	4	5
Never	Rarely	Sometimes	Often	Always

Q5...***how confident did you feel about managing your mycosis fungoides or Sézary syndrome?***

1	2	3	4	5
Absolutely confident	Very confident	Moderately confident	Mildly confident	Not at all confident

Q6. ...***how severe were your mycosis fungoides or Sézary syndrome symptoms?***

1	2	3	4	5	☐
Not at all	A little severe	Somewhat severe	Severe	Very severe	Does not apply (I don't have symptoms right now)

Q7. ... ***how burdensome was your mycosis fungoides or Sézary syndrome treatment?***

1	2	3	4	5	☐
Not at all burdensome	A little burdensome	Somewhat burdensome	Burdensom e	Very Burdensome	Does not apply (I don't have symptoms right now)

Q8. ...***how much did your mycosis fungoides or Sézary syndrome limit your daily activities (work inside and outside of the house, self-care such as cooking, cleaning, getting dressed, etc.)?***

1	2	3	4	5
Not at all	A little bit	Somewhat	Quite a bit	Very much

Q9….***how often did mycosis fungoides or Sézary syndrome (the condition or associated treatment) leave you too tired to work or do daily activities?***				
1<br>Never	2<br>Rarely	3<br>Sometimes	4<br>Often	5<br>Always
Q10. ...***how much did mycosis fungoides or Sézary syndrome negatively affect your relationships with others close to you?***				
1<br>Not at all	2<br>A little bit	3<br>Somewhat	4<br>Quite a bit	5<br>Very much
Q11. ...***how often did you feel that others do not understand what you are going through with mycosis fungoides or Sézary syndrome?***				
1<br>Never	2<br>Rarely	3<br>Sometimes	4<br>Often	5<br>Always
Q12***… to what extent did mycosis fungoides or Sézary syndrome make you feel uncomfortable being around people other than close family and friends?***				
1<br>Never	2<br>Rarely	3<br>Sometimes	4<br>Often	5<br>Always

The following items are not part of the MF/SS-CTCL QoL, but may provide useful information about the patient's experience:

In the past 4 weeks…

...***how much did mycosis fungoides or Sézary syndrome limit your ability to wear clothes you wanted to?***					
1<br>Not at all	2<br>A little bit	3<br>Somewhat	4<br>Quite a bit	5<br>Very much	
…***to what extent were you able to cope with the daily demands (symptom impact and management, treatment, side effects, appointments, etc.) of mycosis fungoides or Sézary syndrome?***					☐<br>Does not apply (I don't have symptoms right now)
1<br>Not at all	2<br>Very little	3<br>Somewhat	4<br>Quite a bit	5<br>To a great extent	

Copyright

The MF/SS-CTCL QoL is licensed under the Creative Commons Attribution-ShareAlike Version 3.0. The full terms of the license are available at http://creativecommons.org/licenses/by-sa/3.0/us/.
